# The effects of telbivudine in late pregnancy to prevent intrauterine transmission of the hepatitis B virus: a systematic review and meta-analysis

**DOI:** 10.1186/1743-422X-9-185

**Published:** 2012-09-04

**Authors:** Min Deng, Xin Zhou, Sheng Gao, Shi-Gui Yang, Bing Wang, Hua-Zhong Chen, Bing Ruan

**Affiliations:** 1 State Key Laboratory for Diagnosis and Treatment of Infectious Diseases, The First Affiliated Hospital, College of Medicine, Zhejiang University, Hangzhou, Zhejiang, 310003, China; 2 Key Laboratory of Combined Multi-Organ Transplantation, The First Affiliated Hospital, College of Medicine, Zhejiang University, Hangzhou, Zhejiang, 310003, China; 3 Department of Infectious Diseases, Taizhou Hospital Affiliated to Wenzhou Medical College, Linhai, Zhejiang, 317000, China

**Keywords:** Hepatitis B virus, Telbivudine, Intrauterine transmission, Pregnanc

## Abstract

Chronic hepatitis B virus (HBV) infection poses a serious public health problem in many parts of the world. Presently, even with proper joint immunoprophylaxis, approximately 10-15% of newborns from HBV carrier mothers suffer from HBV infection through intrauterine transmission. One of the risk factors is the level of maternal viraemia. Telbivudine is a synthetic thymidine nucleoside analogue with activity against HBV. A few studies have evaluated the efficacy of telbivudine in preventing intrauterine HBV infection during late pregnancy. So we conducted this meta-analysis to arrive at an evidence-based conclusion. We searched Medline/PubMed, EMBASE, Cochrane Library, Web of Knowledge and China Biological Medicine Database from January 1990 to December 2011. Relative risks (RR) of the seropositivity rates for hepatitis B surface antigen (HBsAg) and HBV DNA in newborns and infants were studied. Mean differences (MD) in maternal HBV DNA levels were reviewed. Finally two randomised controlled trials (RCTs) and four non-randomised controlled trials (NRCTs) were left for analysis which included 576 mothers in total, of whom 306 received telbivudine treatment and 270 did not receive any drug. All newborns received hepatitis B vaccine (HBVac) and hepatitis B immunoglobulin (HBIG) after birth. The seropositivity rate for HBsAg or HBV DNA was significantly lower in the telbivudine group, both at birth and at 6–12 months follow up. Meanwhile, maternal HBV DNA levels prior to delivery were significantly lower in the telbivudine group. In addition, the frequency of serum creatine kinase (CK) elevation was similar in the two groups. Our meta-analysis provides preliminary evidence that telbivudine application in late pregnancy is effective in the interruption of intrauterine HBV infection, with no significant adverse effects or complications. More high quality, well-designed, double-blinded, randomised controlled and large size clinical trials are needed for further investigation and more convincing results in the future.

## Background

Chronic hepatitis B virus (HBV) infection poses a serious public health problem in many parts of the world, especially in developing countries [1]. In the Asia-Pacific region, vertical transmission of HBV is a key factor in its endemicity. The risk of chronic hepatitis B (CHB) virus infection is inversely proportional to the age at which the infection was acquired [2], and at least 50% of cases acquired the infection during either the perinatal period or in early childhood [3], including through vertical transmission.

Presently, the World Health Organization, the World Gastroenterology Organisation and the Ministry of Health of China recommend joint immunoprophylaxis with the hepatitis B vaccine (HBVac) and hepatitis B immunoglobulin (HBIG) to prevent HBV mother-to-child transmission (MTCT). However, even with proper joint immunoprophylaxis, approximately 10-15% of newborns from HBV carrier mothers suffer from HBV infection through intrauterine transmission [4,5]. Risk factors for HBV immunoprophylaxis failure include maternal hepatitis B e antigen (HBeAg) positivity and increased hepatitis B surface antigen (HBsAg) titre and HBV DNA levels [6]. High maternal serum HBV DNA loads have been reported to increase the possibility of intrauterine HBV infection [7]. Hence, antiviral therapy plus immunoprophylaxis administered to HBV carrier mothers during pregnancy has been suggested to effectively prevent MTCT by reducing maternal HBV DNA levels and developing passive immunisation in the newborns. The oral nucleoside analogue lamivudine is believed to prevent the HBV intrauterine transmission during the last months of pregnancy [8-10]. Many studies have also shown reliable prevention effects of this drug [8,11,12].

Telbivudine, classified as Food and Drug Administration (FDA) pregnancy risk category B [2,3], is a synthetic thymidine nucleoside analogue with activity against HBV. It was reported that telbivudine treatment produced rapid reduction in serum HBV DNA levels in a 4-week dose-escalation trial and a 1-year trial conducted with Asian and Western CHB patients [13]. Besides, global and Chinese clinical trials have indicated that telbivudine provides greater antiviral and clinical efficacy than lamivudine in patients with HBeAg-positive and HBeAg-negative CHB [13,14].

A few studies have evaluated the efficacy of telbivudine in preventing intrauterine HBV infection during late pregnancy. We performed this meta-analysis to arrive at an evidence-based conclusion. We also investigated the safety of telbivudine during pregnancy.

## Methods

### Search strategy

Medline/PubMed, EMBASE, Cochrane Library, Web of Knowledge and China Biological Medicine Database were searched for relevant randomised controlled trials (RCTs) aimed at preventing MTCT of HBV by comparing telbivudine with placebo or no treatment (control). Query results included articles published between January 1990 and December 2011 in English or Chinese peer-reviewed publications (including abstracts). Because published studies were limited, we expanded the search to include non-randomised controlled trials (NRCTs). We also hand-searched bibliographies of reviews, original studies and relevant conference articles, and contacted some investigators directly. The search was designed using the key words “telbivudine or Tyzeka or Sebivo”, “HBV or hepatitis B virus” and “intrauterine or maternity or mother or pregnancy or pregnant”.

### Inclusion and exclusion criteria

Inclusion criteria included the following: (1) maternal blood was positive for HBsAg and HBV DNA; and (2) the diagnoses of intrauterine HBV infection and MTCT were clear; and (3) mothers were HBV carriers or patients with compensated hepatitis [15]. Studies were excluded if they were reviews, meta-analysis or case reports (with no control group). Because HBIG or other antiviral drugs may reduce serum HBV DNA levels, we also excluded studies in which these were used before labour [16]. All newborns (in both the treatment and control groups) received HBIG and HBVac after birth.

### Outcome measures

The primary outcomes were the rates of newborn HBV infection at birth and 6–12 months follow up. Secondary outcomes included maternal blood HBV DNA levels before treatment and prior to delivery, adverse events on the mother (such as elevations in creatine kinase (CK) and aminotransferase (ALT), postpartum haemorrhaging, complications in pregnancy and during delivery), and adverse effects on newborn parameters (such as weight, height and 1-minute Apgar score).

### Study quality

Two investigators (Min Deng and Xin Zhou) independently assessed study quality. All RCTs were graded using the Jadad criteria [17]. These criteria include the three methodological features of randomisation, blinding and accountability. NRCTs had to meet the cases matched by the patient’s characteristics. All studies had defined inclusion and exclusion criteria for patients. Disagreements were resolved by consensus when necessary.

### Data extraction

Extracted data included patient characteristics, design and methods of the study, inclusion and exclusion criteria, treatment duration and dose, primary and secondary outcomes, complications, and adverse events.

### Statistical analysis

Data analysis was carried out with the use of Review Manager Software 5.0 (Cochrane Collaboration). The meta-analysis was performed using the Mantel-Haenszel fixed-effects or random-effects models, depending on the absence or presence of significant heterogeneity [18]. Statistical heterogeneity was assessed by the chi-square (Chi^2^) test and I-squared (I^2^) test and was considered to exist when I^2^ > 50%. We used the relative risk (RR) of the main dichotomous outcomes as the measure of efficacy, which are shown in a forest plot. The 95% confidence interval (CI) for the pooled RR was also provided. Continuous outcomes were presented as a mean difference (MD) because HBV DNA levels of the studies were reported in the same scale (log_10_ copies/ml measured by the Polymerase Chain Reaction). Subgroup analysis was conducted to compare the effects in different study types (RCT or NRCT). A funnel plot estimating the precision of trials was examined to evaluate the potential for selection bias. The overall effect of intervention achieved statistical significance if *P* < 0.05. Because we used only previously published data, approval from the ethics committee was not required.

## Results

### Characteristics of the studies

**Figure 1:**
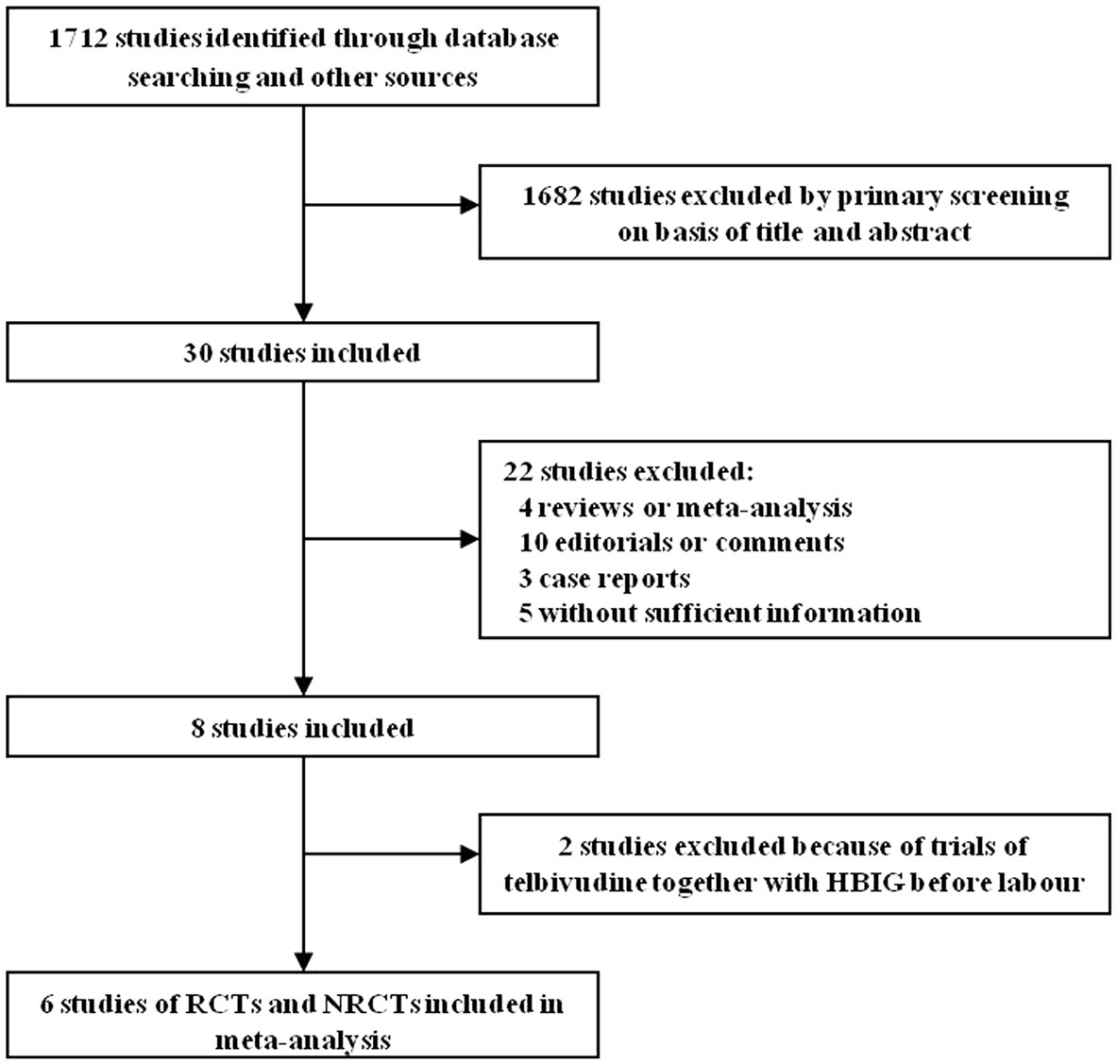
Map of the literature search and selection process.

We identified 1,712 citations through our searches, from which six studies involving 576 mothers (306 of whom were treated with telbivudine) were selected [5,19-23]. A map of the literature search and selection process is shown in Figure 1. Of the studies included, five were published in Chinese [19-23] and one was published in English as original articles [5]. Two studies were RCTs [19,22], but they did not describe the method of randomisation in detail and received Jadad scores of 2. Two studies were not included in our meta-analysis because of the use of telbivudine in combination with HBIG before labour [24,25]. In the trial by Han *et al.*[5], one mother in the telbivudine group had twins, and 10 mothers and their infants (4 in the telbivudine group and 6 in the control group) dropped out at 7 months postpartum. The characteristics of the six included studies are shown in Table 1.

**Table 1:** Characteristics of the included clinical trials in the study

**Study**	**Study design**	**Telbivudine/Control (n)**	**HBeAg (+) (-) (n)**		**Baseline ALT (U/L)**		**HBV DNA level(log copies/ml)**			**Treatment**		**Newborn immunization**	
			**Telbivudine**	**Control**	**Telbivudine**	**Control**	**Telbivudine**	**Control**	**Lower enrollment limit**	**Telbivudine (mg/d)**	**Control**	**HBIG (IU)**	**HVB ac (mg)**
Zhang 2009 [19]	RCT	31/30	NA	NA	NA	NA	Mean (SD): 7.38 (0.81)	Mean (SD): 7,460 (0.45	7	600 from 28-32 wk of gestation to 1 mo after delivery	No treatment	200 at birth and 30 d	10 at 0, 1, 6 mo
Zhao 2010 [22]	RCT	30/30	30/0	30/0	Normal (NA)	Normal (NA)	Positive (NA)	Positive (NA)	Positive (NA)	600 from 28 wk of gestation to 1 mo after delivery	No treatment	200 at birth and 30 d	10 at 0, 1, 6 mo
Zeng 2010 [20]	NRCT	22/26	22/0	26/0	Normal NA)	Normal (NA)	Mean (SD): 7.66 (0.82)	Mean (SD): 7.13 (1.29)	5	600 from 28 wk of gestation to delivery	No treatment	200 at birth	10 at 0, 1, 6 mo
Zhang 2010 [21]	NRCY	60/60	60/0	60/0	NA	NA	Mean (SD): 7.66 (0.82)	Mean (SD): 6.86 (6,10)	6	600 from 28 wk of gestation to 1 mo after delivery	No treatment	200 at birth and 30 d	10 at 0, 1, 6 mo
Yao 2011 [23]	NRCT	28/30	NA	NA	Mean (SD): 93.6 (226.8)	Mean (SD): 50.5 (5.5)	Mean (SD): 7.50 (0.60)	Mean (SD): 7.50 (0.70)	6	600 fro 28 wk of getation to wk after delivery	No treatment	200 at birth	10 at 0, 1, 6H
Han 2011 [5]	NRCT	135/94	135/0	94/0	Mean (SD): 35.67 (43.41	Mean (SD): 42.53 (40.13)	Mean (SD): 8.10 (0.56)	Mean (SD): 7.98 (0.61)	7	600 from 20-32 wk of gestation to 1 mo after delivery	No treatment	200 at birth and 15 d	20 at 0, 1, 6,

RCT: randomized controlled trial; NRCT: non randomized controlled trial: NA: not available; Mean (SD): mean (standard deviation): HBGIG: hepatitis B immunoglobulin; IU: International Unit; HBVac: hepatitis B vaccine.

### Newborn and infant HBsAg seropositivity

**Figure 2:**
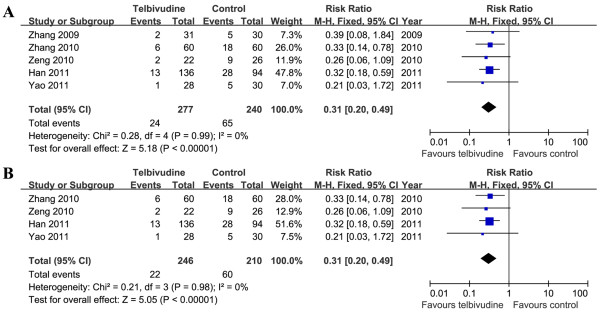
**Forest plots of telbivudine *vs. *control on newborn HBsAg seropositivity.A**. Pooled effect from the combination of RCTs and NRCTs. **B**. Pooled effect from NRCTs.

Five studies (1 RCT and 4 NRCTs) demonstrated newborn HBsAg seropositivity at birth [5,19-21,23]. The newborn HBsAg positive rates were 8.7% (24/277) in the telbivudine group and 27.1% (65/240) in the control group. Based on the Chi^2^ and I^2^ analyses [Chi^2^ = 0.28, df = 4 (*P =* 0.99); I^2^ = 0%], a fixed-effects approach was used to summary estimate the relative risk of telbivudine versus control. The pooled data demonstrated that newborn HBsAg seropositivity was higher in the control group than in the telbivudine group [RR = 0.31, 95% CI (0.20, 0.49), *P* < 0.00001]. We further conducted meta-analysis based on study type (RCT or NRCT) to minimise the potential heterogeneity. However, regarding this parameter, only one RCT [19] could be included. Thus, we pooled the data from the four NRCTs [5,20,21,23]. We found that newborn HBsAg seropositivity was lower in the telbivudine group than in the control group [RR = 0.31, 95% CI (0.20, 0.49), *P* < 0.00001] (Figure 2). The results were consistent.

**Figure 3:**
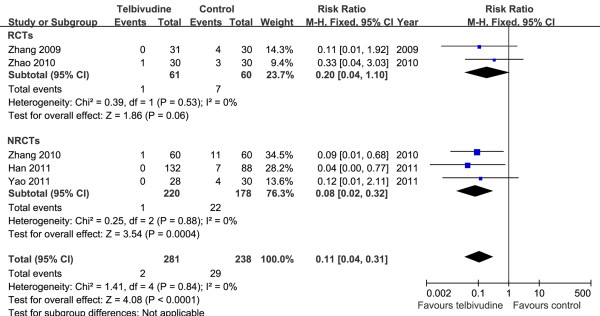
**Forest plots of telbivudine *vs. *control on infant HBsAg seropositivity at age 6–12 months.**

At age 6–12 months, the infant HBsAg positive rates were 0.7% (2/281) in the telbivudine group and 12.2% (29/238) in the control group (2 RCTs and 3 NRCTs) [5,19,21-23]. Based on the Chi^2^ and I^2^ analyses, significant differences in heterogeneity were not observed between the two groups [Chi^2^ = 1.41, df = 4 (*P =* 0.84); I^2^ = 0%]. A summary estimate of the relative risk of telbivudine versus control showed a significant difference [RR = 0.11, 95% CI (0.04, 0.31), *P* < 0.0001]. We also conducted meta-analysis for two RCTs [19,22] and three NRCTs [5,21,23]. The pooled RR of the two RCTs was 0.20 [95% CI (0.04, 1.10)]. Although it did not achieve statistical significance (*P* = 0.06), there was a trend towards a decrease in HBsAg positive rate in infants in the telbivudine group. The pooled results of the three NRCTs showed that the RR was 0.08 [95% CI (0.02, 0.32), *P* = 0.0004] (Figure 3).

**Figure 4:**
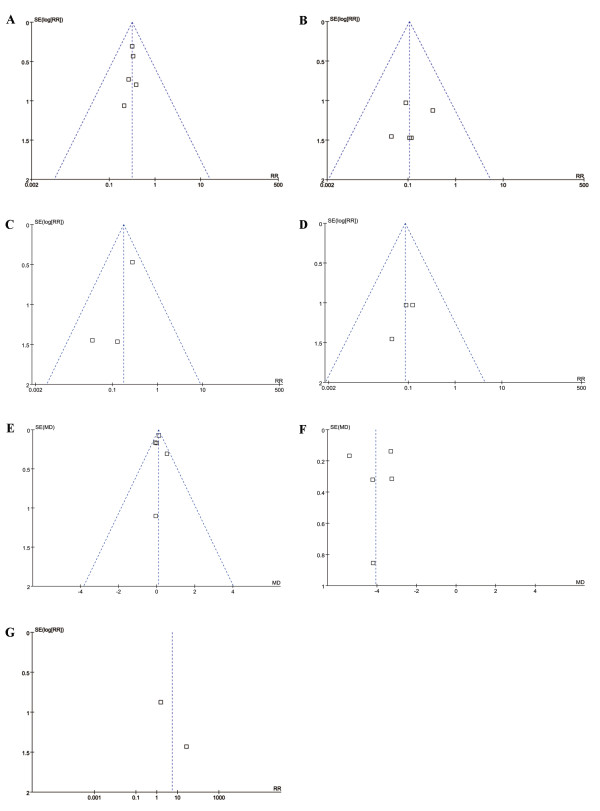
**Funnel plot analysis to detect selection bias.A**. Funnel plots of studies on newborn HBsAg seropositivity. **B**. Funnel plots of studies on infant HBsAg seropositivity. **C**. Funnel plots of studies on newborn HBV DNA seropositivity. **D**. Funnel plots of studies on infant HBV DNA seropositivity. **E**. Funnel plots of studies on maternal blood HBV DNA levels before treatment. **F**. Funnel plots of studies on maternal blood HBV DNA levels prior to delivery. **G**. Funnel plots of studies on elevation of maternal serum creatine kinase.

We further explored the selection biases, and found no significant asymmetry of the funnel plots (Figure 4). The immunoprophylaxis outcomes for newborns and infants are shown in Table 2.

**Table 2:** The immunoprophylaxis outcomes for newborns and infants

**Study**	**Newborns number**	**HBsAgseropositivity n (%)**								**HBV DNA seropositivity n (%)**							
	**Telbivudine/Control**	**Telbivudine/**				**Control**				**Telbivudine**				**Control**			
	**n**	**At birth**	**6 mo**	**7 mo**	**12 mo**	**At birth**	**6 mo**	**7 mo**	**12 mo**	**At birth**	**6 mo**	**7 mo**	**12 mo**	**At birth**	**6 mo**	**7 mp**	**12 mo**
Zhang 2009 [21]	31/30	2 (6.5%)	NA	0 (0%)	NA	5 (16.7%)	NA	4 (13.3%)	NA	NA	NA	NA	NA	NA	NA	NA	NA
Zhao 2010 [22]	30/30	NA	NA	NA	1 (3.3%)	NA	NA	NA	3 (10.0%)	NA	NA	NA	1 (3.3%)	NA	NA	NA	8 (26.7%)
Zeng 2010 [20]	22/26	2 (9.1%)	NA	NA	NA	9 (34.6%)	NA	NA	NA	0 (0%)	NA	NA	NA	4 (15.4%)	NA	NA	NA
Zhang 2010 [21]	60/60	6 (10.0%)	NA	NA	1 (1.7%)	18 (30.0%)	NA	NA	11 (18.3%)	5 (18.3%)	NA	NA	1 (1.7%)	18 (30.0%)	NA	NA	11 (18.3%)
Yao 2011 [23]	28/30	1 (3.6%)	0 (0%)	NA	NA	5 (16.7%)	4 (13.3%)	NA	NA	NA	NA	NA	NA	NA	NA	NA	NA
Han 2011 [5]	136/94	13 (9.6%)	NA	0 (0%)	NA	28 (29.8%)	NA	7 (8.0%)	NA	0 (0%)	NA	0 (0%)	NA	9 (9.6%)	NA	7 (8.0%)	NA

HBsAg: hepatitis B surface antigen; NA: not available.

### Newborn and infant HBV DNA seropositivity

**Figure 5:**
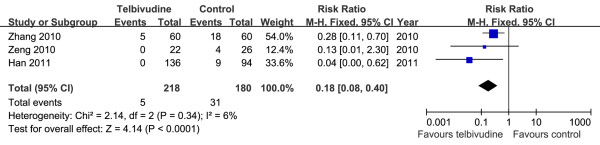
**Forest plots of telbivudine *vs. *control on newborn HBV DNA seropositivity.**

Only three NRCTs examined in this meta-analysis reported HBV DNA seropositivity at birth [5,20,21]. The newborn HBV DNA positive rates were 2.3% (5/218) in the telbivudine group and 17.2% (31/180) in the control group. Using the Chi^2^ and I^2^ analyses, heterogeneity was assessed and not found to be significant [Chi^2^ = 2.14, df = 2 (*P =* 0.34); I^2^ = 6%]. The pooled data of the three studies showed a significant difference between the two groups [RR = 0.18, 95% CI (0.08, 0.40), *P* < 0.0001] (Figure 5).

**Figure 6:**
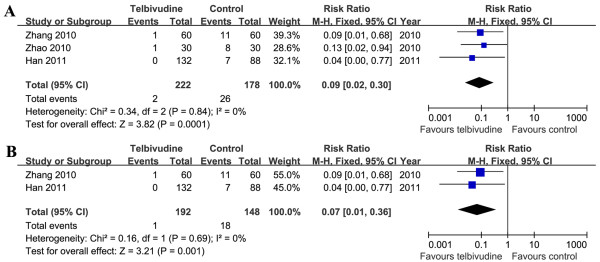
**Forest plots of telbivudine *vs. *control on infant HBV DNA seropositivity at age 6–12 months.A**. Pooled effect from the combination of RCTs and NRCTs. **B**. Pooled effect from NRCTs.

At age 6–12 months, three trials (1 RCT and 2 RCTs) demonstrated infant HBV DNA seropositivity [5,21,22]. The infant HBV DNA positive rates were 0.9% (2/222) in the telbivudine group and 14.6% (26/178) in the control group. According to Chi^2^ and I^2^ analyses, significant differences in heterogeneity were not observed [Chi^2^ = 0.34, df = 2 (*P =* 0.84); I^2^ = 0%]. A summary estimate of the relative risk of telbivudine versus control using a fixed-effects approach demonstrated that there was a significant difference in infant HBV DNA seropositivity between two groups [RR = 0.09, 95% CI (0.02, 0.30), *P* = 0.0001]. As only one RCT [22] could be included in this meta-analysis, we pooled the data from the two NRCTs [5,21]. We found that the results were consistent [RR = 0.07, 95% CI (0.01, 0.36), *P* = 0.001] (Figure 6).

We further explored the selection biases of the outcomes, and found no significant asymmetry of the funnel plots (Figure 4).

### Maternal blood HBV DNA levels

**Figure 7:**
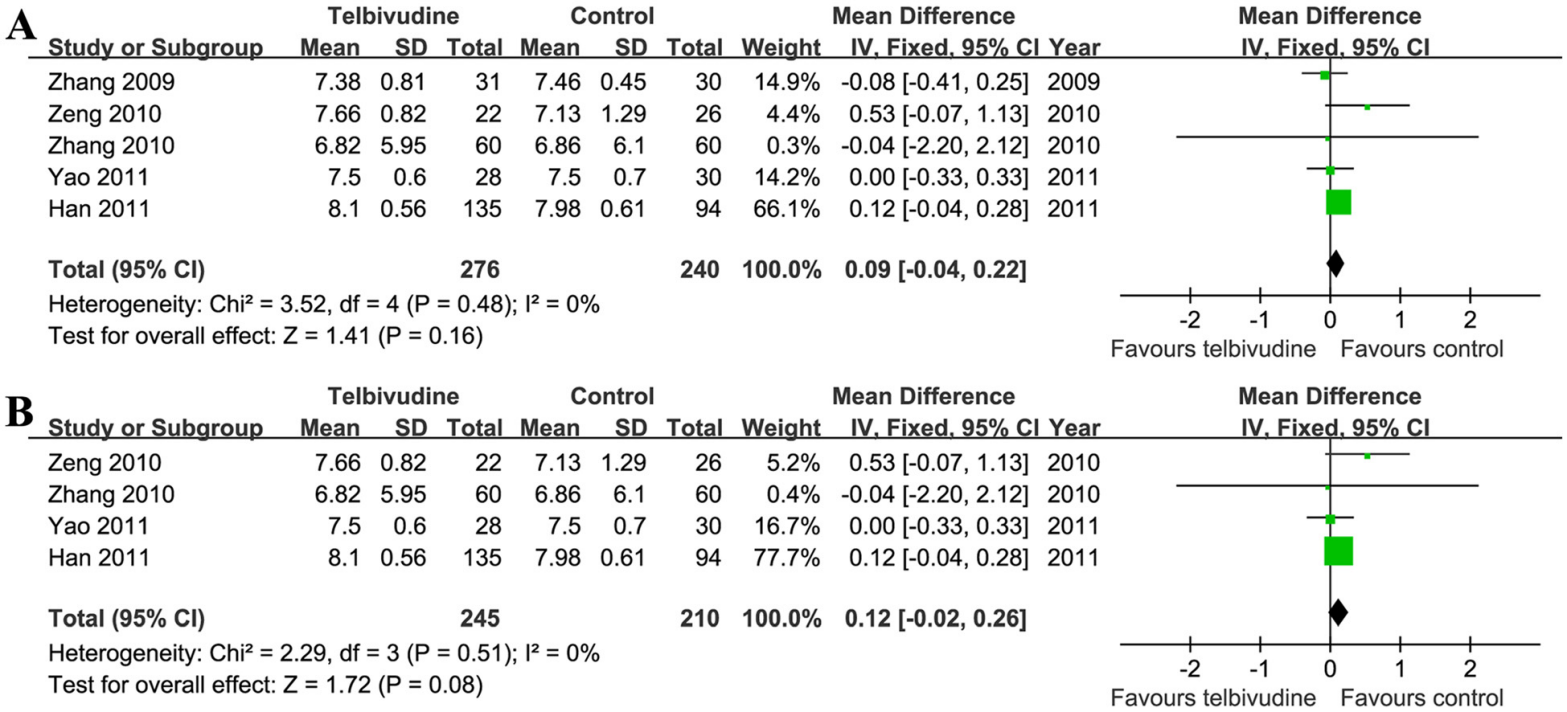
**Forest plots of telbivudine *vs. *control on maternal blood HBV DNA levels before treatment.A**. Pooled effect from the combination of RCTs and NRCTs. **B**. Pooled effect from NRCTs.

Five studies (1 RCT and 4 NRCTs) described maternal blood HBV DNA levels before telbivudine treatment in detail [5,19-21,23]. Heterogeneity was assessed and not found to be significant [Chi^2^ = 3.52, df = 4 (*P =* 0.48); I^2^ = 0%]. A summary estimate of the MD of telbivudine versus control using a fixed-effects approach demonstrated that maternal blood HBV DNA levels were similar before telbivudine treatment [MD = 0.09, 95% CI (−0.04, 0.22), *P* = 0.16]. As we had to exclude one RCT [19], we conducted a meta-analysis for the four NRCTs [5,20,21,23]. The pooled data also showed no significant difference in maternal blood HBV DNA levels before treatment [MD = 0.12, 95% CI (−0.02, 0.26), *P* = 0.08] (Figure 7).

**Figure 8:**
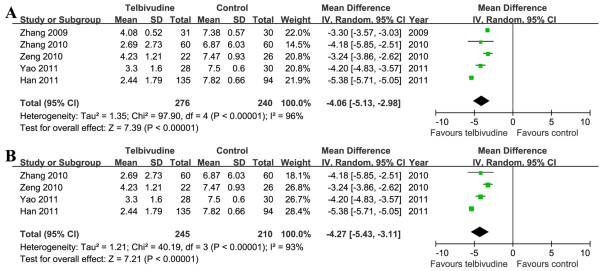
**Forest plots of telbivudine *vs. *control on maternal blood HBV DNA levels prior to delivery.A**. Pooled effect from the combination of RCTs and NRCTs. **B**. Pooled effect from NRCTs.

Also among the five studies, Chi^2^ and I^2^ analyses identified significant heterogeneity in maternal blood HBV DNA levels between two groups prior to delivery [Tau^2^ = 1.35, Chi^2^ = 97.90, df = 4 (*P <* 0.00001); I^2^ = 96%]. A summary estimate of the MD of telbivudine versus control using a random-effects approach demonstrated that the maternal blood HBV DNA level was lower in the telbivudine group prior to delivery [MD = −4.06, 95% CI (−5.13, -2.98), *P <* 0.00001]. We also further performed a meta-analysis among the four NRCTs, and the results were consistent [MD = −4.27, 95% CI (−5.43, -3.11), *P* < 0.00001] (Figure 8).

In addition, we explored the selection biases, and found no significant asymmetry of the funnel plots (Figure 4).

### Safety

Elevation of serum CK in newborns (8 in the telbivudine group and 9 in the control group) was the only adverse event reported by Zhang *et al.*[19]. Thirteen mothers presented with elevated CK levels in another study by Zhang *et al.*[21]. The only reported adverse event in Yao’s trial [23] was elevated CK levels in 3 mothers in the telbivudine group and 2 mothers in the control group. In addition, 5 mothers in Zeng’s study [20] and 88 mothers in Han’s study [5] had light elevation in serum ALT levels after drug withdrawal. At 7 month follow up, pneumonia and other adverse events in infants were evaluated and considered not to be drug related in Han’s study.

**Figure 9:**
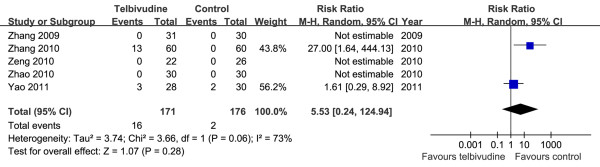
**Forest plots of telbivudine *vs. *control on elevation of maternal serum creatine kinase.**

Fives studies (2 RCTs and 3 NRCTs) examined in this meta-analysis reported serum CK levels in mothers [19-23]. According to Chi^2^ and I^2^ analyses [Tau^2^ = 3.74, Chi^2^ = 3.66, df = 1 (*P =* 0.06); I^2^ = 73%], a random-effects approach was used to summary estimate the relative risk of telbivudine versus control. The pooled data demonstrated that the frequency of serum CK elevation was similar in the two groups [RR = 5.53, 95% CI (0.24, 124.94), *P* = 0.28] (Figure 9). Because two studies substantially contributed to the overall effect of the meta-analysis, we did not divide the studies into subgroups to investigate heterogeneity.

We further explored the selection bias, and found no significant asymmetry of the funnel plots (Figure 4).

## Discussion

Of the estimated 350 million individuals chronically infected with HBV worldwide, it is generally accepted that at least 50% of cases acquired their infections during either the perinatal period or in early childhood, especially in areas of high endemicity. In China, where the prevalence of HBV is high, 85-90% of transmissions can be prevented successfully with proper immunoprophylaxis [5]. However, approximately 10-15% of newborns from HBV carrier mothers suffer from HBV infection because of intrauterine transmission. MTCT, which includes intrauterine transmission, perinatal transmission and transmission during lactation, is an important reason for failure of immune prophylaxis [26,27]. It has been demonstrated that active immunisation (HBVac) combined with passive immunisation (HBIG) can effectively prevent perinatal transmission and transmission during lactation [26,28,29]. Nonetheless, the incidence of intrauterine HBV infection remains as high as 43% when maternal serum HBV DNA exceeds 10^8^ copies/ml [30], even with prompt administration of active and passive vaccination. del Canho *et al.* also reported that intrauterine infection occurs only when maternal serum HBV DNA levels are high (> 150 pg/ml or 3.16 × 10^7^ copies/ml) [31]. Intrauterine HBV transmission occurs primarily during the third trimester [32] and is presumed to cause a minority of the infections not prevented by prompt immunisation. Probable reasons for transmission are thought to be the breach of the placental barrier caused by HBV [3] and polymorphisms in some cytokine genes (such as those encoding for interferon-γ and tumour necrosis factor-α) [33].

In general, it is not recommend to initiate antiviral therapy with nucleoside analogues in HBV-infected persons who are immune tolerant. However, they have the same risk of intrauterine infection as CHB patients for their high HBV DNA levels. Because the risk of HBV intrauterine infection is clearly related to the level of maternal viraemia, and most foetal organs have developed by the third trimester, a strategy to interrupt this process is maternal treatment with antiviral drug in late pregnancy. Interferon and peg-interferon are contraindicated during pregnancy largely because of their known anti-proliferative effects. Lamivudine, the first nucleoside analogue inhibitor to be approved for the treatment of CHB, has long been used for both HIV and HBV infection during pregnancy and has displayed reliable prevention effects [34]. Even though lamivudine is classified as FDA pregnancy risk category C, the European Association for the study of the Liver also confirmed its safety during late pregnancy in 2009 [35]. However, it was also reported that reducing maternal HBV DNA levels even to undetectable status by lamivudine in late pregnancy could not guarantee prevention of intrauterine HBV infection in the newborns [36]. Telbivudine was approved by the FDA in 2006 and the State Food and Drug Administration in 2007 for the treatment of patients with CHB, and has demonstrated faster and better efficacy than lamivudine in patients with HBeAg-positive and HBeAg-negative CHB disease.

A few studies have evaluated the efficacy of telbivudine application in preventing intrauterine HBV infection during late pregnancy. Therefore we conducted this meta-analysis to arrive at an evidence-based conclusion. In this meta-analysis, the treatment group consisted of 306 mothers who received 600 mg/d telbivudine from the second or third trimester of pregnancy until delivery or 1 month after delivery. The control group consisted of 270 mothers who did not receive any antiviral drug. All newborns received HBIG and HBVac after birth. The pooled results clearly showed that the seropositivity rate for HBsAg or HBV DNA was significantly lower in the telbivudine group, both at birth and at 6–12 months follow up. Meanwhile, maternal HBV DNA levels prior to delivery were significantly lower in the telbivudine group. We further conducted meta-analysis based on study type (RCT or NRCT) to minimise any potential heterogeneity and found that the type of study design did not affect the conclusion. However, we should pay more attention to the evidence grades of these included trials. Because published studies were limited, only six trials were included in the meta-analysis. Two trials contained randomised controls, but the details of the randomisation sequence were not provided. The other four trials included non-randomised controls. In the Han [5], Zeng [20] and Zhang, 2010 [21] studies, the mothers self-selected themselves into one arm or the other. When some demographic features were presented, more of the control group had abnormal ALT levels [5]. In Yao’s study [23], the mothers were divided into different groups according to baseline ALT levels. Thus, more high-quality, well-designed and randomised controlled multi-centre trails are necessary to verify the results in the future.

As an orally bioavailable L-nucleoside analogue with potent activity against HBV, telbivudine has shown no effects on human nucleotides and DNA synthesis [37]. Toxicology research has also demonstrated that telbivudine has no significant organ toxicity, carcinogenicity, genotoxicity, mitochondrial toxicity *in vitro*, teratogenicity, or embryo-foetal toxicity [13,38]. In the phase III GLOBE trial, most adverse events reported were classified as mild or moderate in severity and were not attributed to telbivudine, and elevations in serum CK were more common in the telbivudine group. Based on its characteristics, telbivudine is listed by the FDA as a pregnancy category B drug. The treatment guidelines of the Asian Pacific Association for the Study of the Liver also designated telbivudine as a replacement antiviral therapy during pregnancy in 2008 [39]. In our meta-analysis we analysed elevations in serum CK, elevated ALT levels after drug withdrawal and other adverse events. Since the reported studies only evaluated the short-term effects of telbivudine and only a few studies reported adverse events, the long-term safety of telbivudine in infants still need to be assessed in the future. In addition, we found that the number of infected infants at age 6–12 months was less than the number of infected newborns. The probable reason for this decline is that maternal blood containing HBsAg and HBV DNA was introduced into the bodies of the newborns through the umbilical cord during delivery, which were later neutralised by anti-HBs of HBIG [40].

To our knowledge, this is the first systematic meta-analysis of this topic. We are convinced that our search strategy was comprehensive and exhaustive. We comply with the standardized guidelines on the reporting of systematic reviews according to the PRISMA statement and the PRISMA checklist (see Additional file 1). Two investigators (Min Deng and Xin Zhou) independently extracted data and entered them in a customised form. Disagreements were resolved by consensus when necessary. However, the following limitations of our meta-analysis should be considered. First, we searched for studies published only in English or Chinese. Second, due to limited published studies, we expanded the search to include NRCTs. Third, only a few studies were included and they had small sample sizes. For these reasons, we could not perform a deep analysis. Besides, all included studies were performed in mainland China. Therefore, more clinical studies performed in different populations and other regions are necessary to access the generalisability of the results.

## Conclusions

Our meta-analysis provides preliminary evidence that telbivudine application in late pregnancy is effective in the interruption of intrauterine HBV infection, with no significant adverse effects or complications. More high quality, well-designed, double-blinded, randomised controlled and large size clinical trials are needed for further investigation and more convincing results in the future.

## Abbreviations

HBV: Hepatitis B virus; RR: Relative risk; HBsAg: Hepatitis B surface antigen; MD: Mean difference; RCTs: Randomized controlled trials; NRCTs: Non-randomized controlled trials; HBVac: Hepatitis B vaccine; HBIG: hepatitis B immunoglobulin; CK: Creatine kinase; CHB: Chronic Hepatitis B; MTCT: Mother-to-child transmission; HBeAg: Hepatitis B e antigen; FDA: the Food and Drug Administration; ALT: Aminotransferase; CI: Confidence interval.

## Competing interests

The authors declare that they have no competing interests.

## Authors’ contributions

RB conceived the study and revised the manuscript critically for important intellectual content. DM and ZX made substantial contributions to its design, acquisition, analysis and interpretation of data. GS, YSG, WB and CHZ participated in the design, acquisition, analysis and interpretation of data. All authors read and approved the final manuscript.

## Supplementary Material

Additional file 1 PRISMA 2009 Checklist. Click here for file
